# The Asymmetrical Structure of Golgi Apparatus Membranes Revealed by *In situ* Atomic Force Microscope

**DOI:** 10.1371/journal.pone.0061596

**Published:** 2013-04-16

**Authors:** Haijiao Xu, Weiheng Su, Mingjun Cai, Junguang Jiang, Xianlu Zeng, Hongda Wang

**Affiliations:** 1 State Key Laboratory of Electroanalytical Chemistry, Changchun Institute of Applied Chemistry, Chinese Academy of Sciences, Changchun, Jilin, P.R. China; 2 Institute of Genetics and Cytology, Northeast Normal University, Changchun, China; 3 National engineering laboratory for AIDS vaccine, College of Life Science, Jilin University, Changchun, China; National Cancer Institute, United States of America

## Abstract

The Golgi apparatus has attracted intense attentions due to its fascinating morphology and vital role as the pivot of cellular secretory pathway since its discovery. However, its complex structure at the molecular level remains elusive due to limited approaches. In this study, the structure of Golgi apparatus, including the Golgi stack, cisternal structure, relevant tubules and vesicles, were directly visualized by high-resolution atomic force microscope. We imaged both sides of Golgi apparatus membranes and revealed that the outer leaflet of Golgi membranes is relatively smooth while the inner membrane leaflet is rough and covered by dense proteins. With the treatment of methyl-β-cyclodextrin and Triton X-100, we confirmed the existence of lipid rafts in Golgi apparatus membrane, which are mostly in the size of 20 nm –200 nm and appear irregular in shape. Our results may be of significance to reveal the structure-function relationship of the Golgi complex and pave the way for visualizing the endomembrane system in mammalian cells at the molecular level.

## Introduction

The Golgi apparatus is a key organelle of the endomembrane system, locating at the pivot of the classical secretory pathway. Typically, the Golgi apparatus consists of a series of flattened cisternal membranes that are in parallel to form a stack with periphery vesicles and tubules [1]. The Golgi apparatus is a dynamic organelle, responsible for receiving, processing, and sorting newly synthesized proteins and lipids through the secretory pathway [2]. Recent evidences indicated that the elaborate Golgi apparatus is also associated with signal transduction [3], [4]. Besides, it is widely assumed that ER-Golgi network may become a future target for anti-cancer therapy [5].

The proteins in Golgi membranes are the basis for the Golgi apparatus to perform important intracellular functions, such as membrane sorting, membrane traffic and signal transduction. Therefore, studying the protein distribution of Golgi membranes is of significance to reveal their functions at the molecular level. In addition, biological membranes consisting of various lipids and proteins are not homogeneous [6], [7], which is considered as a requirement to perform its functions [8], [9]. Membrane lateral heterogeneity is usually termed as “lipid rafts” that are dynamic microdomains enriched with cholesterol, sphingolipids and proteins [10], [11]. It is reported that in mammalian cells lipid rafts are first assembled in the Golgi complex where sphingolipids are synthesized [11], [12].

The structure of Golgi apparatus has been the focus of biologists since its original description in 1898 [13]–[15]. To date, the major approaches to study the Golgi apparatus are electron microscopy (EM) and light microscopy [1], [16], [17]. However, the disadvantage of those methods is the limitation of the spatial resolution of light microscopy, as well the inability of EM to real-time analyze and image biology samples under physiological conditions. Therefore, the direct investigation of Golgi apparatus structure at the molecule level is not achieved. Although studies on the lipid rafts of the Golgi membranes have been reported [18]–[20], direct detection of lipid rafts in the Golgi membranes is still a challenge due to their small size and dynamic property. Due to the complicated structure of the Golgi apparatus, disclosing the relationship of its structure-function at the molecule level necessarily depends on advanced experimental methods. Therefore, it is of importance to explore new experimental approaches with the capability of directly imaging the Golgi membranes under near-native conditions.

With the advantage of imaging biological samples in solution without fixation and staining, atomic force microscopy (AFM) has been a powerful tool in biological researches. AFM has been demonstrated to study the surface topography of biological membranes at a high resolution [21], [22]. Meanwhile, owing to the ability to real-time image biological samples, AFM has studied lipid rafts in model membranes [23]. Recently, our results have successfully confirmed the existence of lipid rafts in human erythrocytes membranes by in-situ and time-lapse AFM [21]. The development of time-lapse AFM solves the difficulty to understand the dynamic characteristics of the biological molecules, thus providing an efficient tool to approach the relationship between their structure and functions. Herein, we utilized in-situ AFM to image the isolated Golgi membrane fractions stably attached onto the APTES-mica surface under quasi-native conditions, revealing the asymmetrical structure of the Golgi membranes.

## Results

### AFM Image of Golgi Apparatus

The Golgi membrane fractions were prepared by sucrose density gradient centrifugation. The isolated Golgi membrane fractions was first confirmed by Western blot analysis using anti-β-1,4-Galactosyltransferase antibody (Fig. S1). To further verify the existence of the Golgi fractions in isolated samples, the samples were treated with Golgi-tracker Red (a specific fluorescent dye of the Golgi complex) and imaged by fluorescence microscopy (Fig. S2).

**Figure 1:**
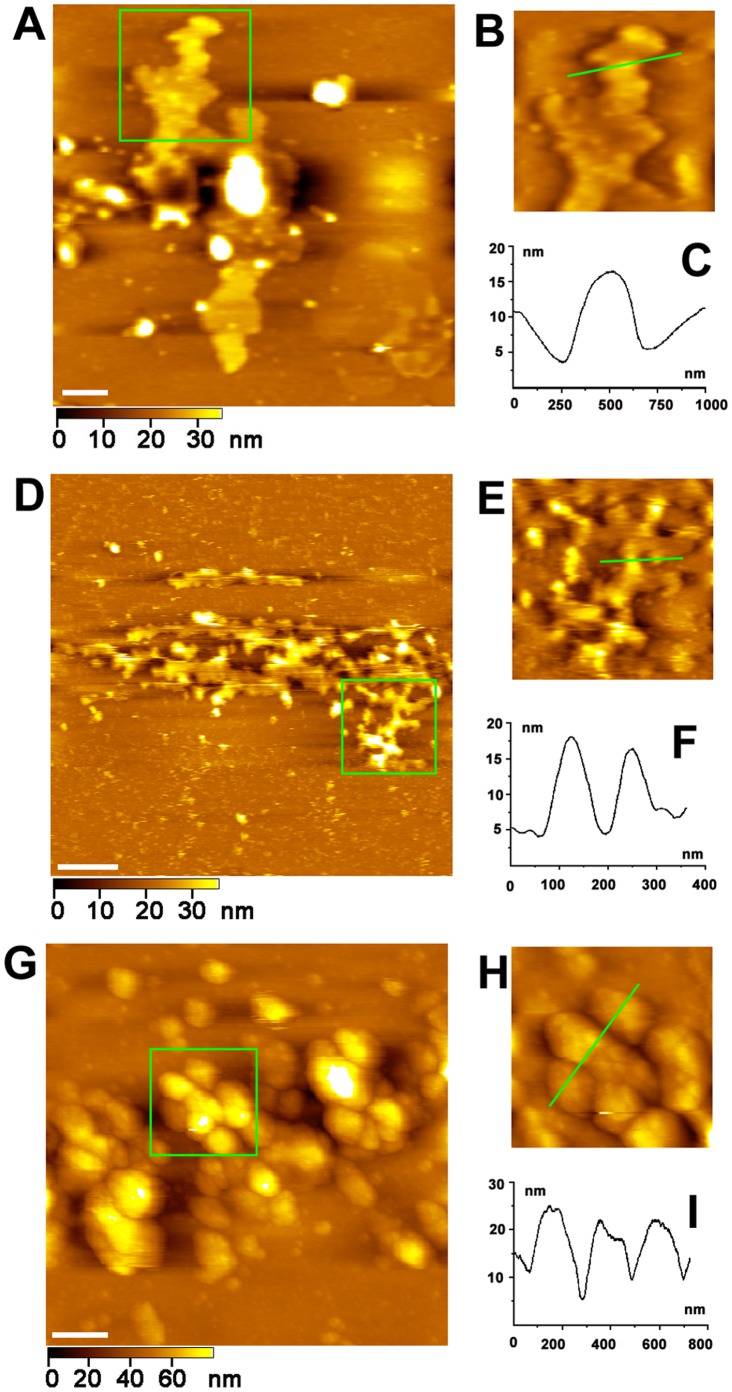
AFM images of Golgi apparatus. (A) AFM image of a stack of membranous cisternae. Scale bar is 1 µm. (B) Magnification of the green square area in (A). (C) Cross-section analysis along the green line drawn in (B). (D) AFM image of tubule network of Golgi apparatus. Scale bar is 1 µm. (E) Magnification of the green square area in (D). (F) Cross-section analysis along the green line drawn in (E). (G) AFM image of vesicles of Golgi apparatus. Scale bar is 1 µm. (H) Magnification of the green square area in (G). (I) Cross-section analysis along the green line drawn in (H).

To obtain the detailed information about the Golgi membrane fractions, we imaged them by AFM under near-native conditions. Fig. 1A shows the AFM topographical image of the Golgi stack consisting of two closely interconnected Golgi cisternae. A few round Golgi cisternae are also observed. The representative Golgi compartments tubules are shown in Fig. 1D. Generally, the tubules are in the formation of the tubular-reticular networks, which are responsible for the Golgi organization, stack connection, and cargo transport [24], [25]. In the Fig. 1G, the continuous grape-like strings of vesicles with the size of 50–60 nm are observed. As reported, these vesicles could serve as carriers for cargo transportation and Golgi enzymes recycling [26], [27]. As reported, the Golgi apparatus consists of three main compartments, including flat disc-shaped cisternae, associated abundant tubular-reticular networks and vesicles [28], [29]. Our observation in Fig. 1 is completely consistent with the classical description, and provides more direct and concrete details.

To distinguish the Golgi morphology from the other cellular compartments, we have further achieved the AFM imaging of the cell membrane, mitochondria and endoplasmic reticulum (Fig. S3). It is found that there are apparent differences between the Golgi apparatus and the other cellular organelles in morphology.

### AFM Image of Individual Golgi Cisternae

**Figure 2:**
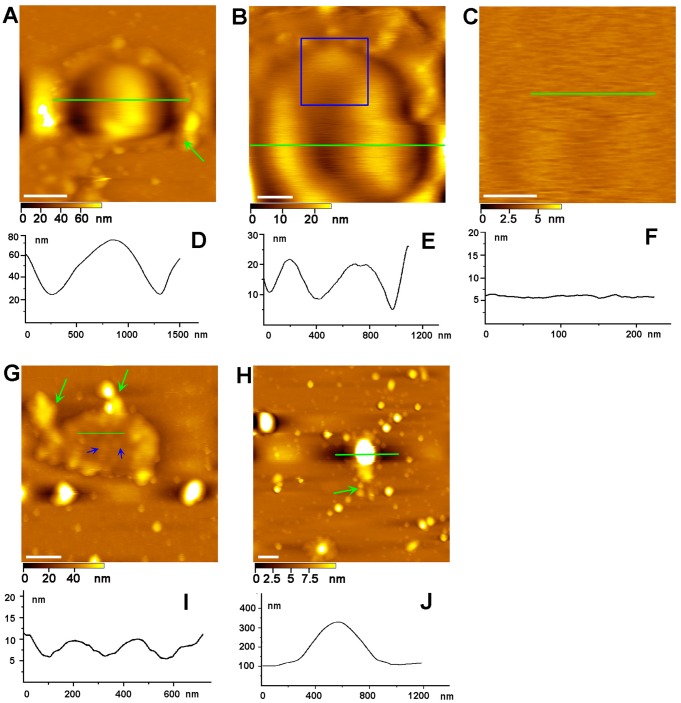
AFM image of individual Golgi cisternae. (A) AFM Image of a globular membranous cisterna. Scale bar is 400 nm. (B) Magnification of the cisterna in (A). Scale bar is 200 nm. (C) Higher magnification of the blue boxed area in (B). Scale bar is 100 nm. (D, E, F) Cross section analysis along the green line drawn in (A) (B) and (C). (G) AFM image of a flat membranous cisterna. Blue arrows point to protein particles underneath the cisterna. Scale bar is 500 nm. (H) AFM image of an oval-shaped membranous cisterna with peripheral vesicles. Scale bar is 500 nm. (I and J) Cross section analysis along the green line drawn in images (G) and (H). Green arrows in Fig. 2 point to buds or interconnected vesicles around the cisterna.

The Golgi apparatus is typically composed of a series of stacked flat cisternae. Single cisterna as a basic unit plays an important role in Golgi’s functions. As well known, the cisternae display diverse shapes with budding vesicles surrounding their outer edges [28], [30]. As observed in Fig. 2A, the globular membranous cisterna includes a smooth-surfaced central area with about 900 nm in diameter and a flat peripheral area with coated buds, as previously described [16]. Fig. 2B shows the magnified image in Fig. 2A. Apparently, the smooth central area is depressed likely caused by tip pressure during scanning, indicating that the central area is relatively soft. The plasticity of the Golgi membranes may shed light on the question why the Golgi apparatus is highly dynamic. To further observe the surface of the cisterna, the higher resolution image (the blue box area in Fig. 2B) was achieved, as shown in Fig. 2C. The average roughness of the outer membrane leaflet of the cisterna was only about 0.43±0.09 nm, which demonstrates that the membrane surface is extremely smooth.

Fig. 2G shows a topographic image of a flat individual cisterna with buds. Some blurry protrusions can be observed as pointed by blue arrows, possibly representing protein particles underneath the cisterna surface as a result of AFM tip pressure. Fig. 2H shows an oval-shaped cisterna with multiple buds and vesicles. The AFM images reveal that these cisternae possess some common features including smooth membrane surfaces, buds or interconnected vesicles located at margins as indicated by green arrows in Fig. 2.

### Observation of the Inner Leaflet of Golgi Membranes

**Figure 3:**
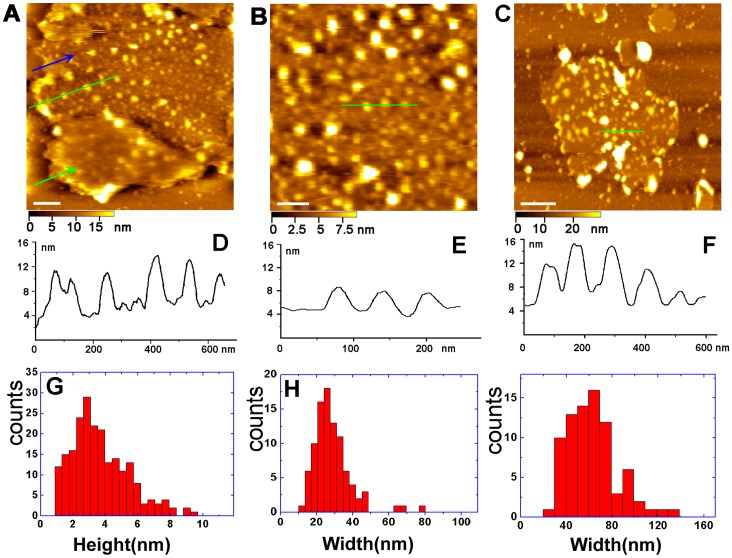
AFM image of the inner leaflet of Golgi membrane. (A) Image of an opened cisterna. Blue and green arrows point to the inner and outer leaflet membranes, respectively. Scale bar is 500 nm. (B) Magnification of the inner membrane leaflet membrane in (A). Scale bar is 100 nm. (C) The inner membrane leaflet of the Golgi cisterna membrane. Scale bar is 500 nm. (D–F) Cross-section analysis along the lines in images (A–C), respectively. (G) Height distribution of proteins in the inner leaflet membrane in (A). (H) Width distribution of proteins in the inner membrane leaflet in (B). (I) Width distribution of proteins in the inner leaflet membrane in (C).

To observe the inner leaflet of Golgi membranes in its quasi-native state, the isolated Golgi membrane fractions adsorbed on APTES-mica in PBS solution were imaged by in-situ AFM. Fig. 3A represents a typical image of a half-opened Golgi cisterna caused by AFM tip [31], [32]. The Golgi cisterna was tightly attached on the surface of APTES-mica, exposing the inner membrane leaflet with dense proteins and the outer leaflet of the Golgi membrane as marked by blue arrow and green arrow, respectively (Fig. 3A). The outer leaflet membrane also displays the outline of the proteins underneath the membranes, possibly due to the AFM tip pressure.

The average height of inner leaflet membrane between the mica substrate and the proteins was about 7 nm. The height of pure lipid bilayer under the membrane proteins are about 3.73±1.60 nm, which is consistent with reported result [33]. The average roughness of the inner leaflet membrane was about 1.95±0.62 nm. To further observe the inner membrane leaflet in detail, the higher resolution image was achieved (Fig. 3B), where the proteins are close to each other. These proteins were mostly embedded in the inner membrane of Golgi cisternae, which is very similar with the protein distribution in red blood cell membranes [22].

The proteins in the inner membrane leaflet have a broad height distribution between 1 nm and 10 nm with the multiple peaks (Fig. 3G). Moreover, these proteins display a broad mean diameter distribution between 10 nm and 80 nm, as shown in Fig. 3H. We assumed that the large sized particles are attributed to proteins aggregates necessary to perform physiological functions.

To clarify the heterogeneity of different Golgi membranes, we imaged different Golgi membrane fractions. Fig. 3C represents an inside-out Golgi membrane covered by proteins. It is evident that the proteins are much larger in size than the proteins imaged in the Fig. 3B, showing a mean diameter distribution between 20 nm and 140 nm as indicated in Fig. 3I. The smooth regions among the proteins particles are considered possibly as free lipid bilayer with the average height of about 5 nm, which indicates that some transmembrane proteins might be embedded in lipid bilayer.

### The Existence of Lipid Rafts in Golgi Membranes

**Figure 4:**
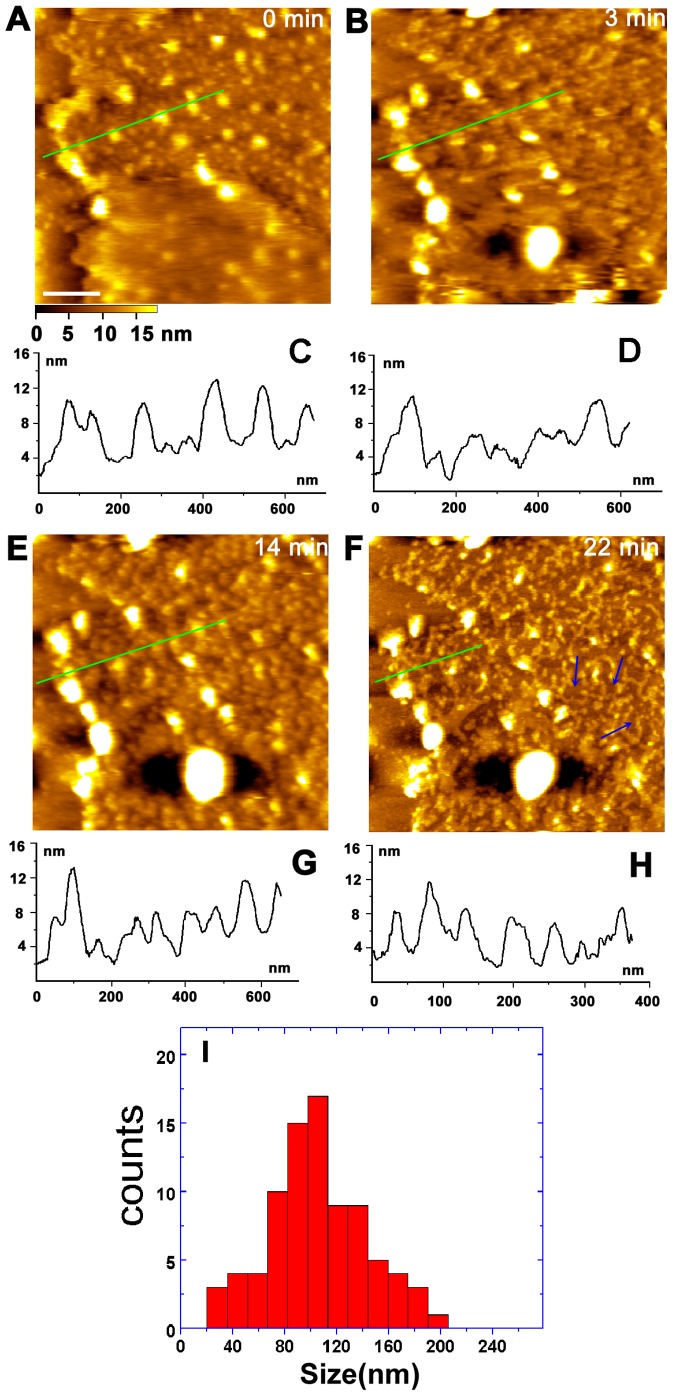
Real-time images of Golgi membrane treated with MβCD. (A,B,E,F) Series of images after the treatment with MβCD for 0 min (A), 3 min (B), 14 min (E), 22 min (F). Blue arrows in (F) point to the regions of the Golgi membrane eroded by MβCD. Cross-section analyses of images (A, B, E, F) are shown in (C, D, G, H), respectively. (I) Size distribution of the regions of the Golgi membrane eroded by MβCD in Fig. 4F. Scale bar in (A) is 200 nm.

Cholesterol is an essential component of eukaryotic cell membranes and plays a crucial role in the assembly and maintenance of sphingolipid-rich rafts [34], [35], thus its depletion can result in the dissolution of lipid rafts. The cholesterol-sequestering agent, methyl-β-cyclodextrin (MβCD), has been widely used to disrupt lipid rafts in cellular studies by extracting cholesterol [36], [37]. To confirm the existence of lipid rafts, MβCD was in situ injected into the AFM sample chamber, and successive real-time AFM images in the same area were recorded to monitor the changes (Fig. 4).

Fig. 4A depicts an AFM image of a half-opened Golgi cisternae with dense proteins. After the injection of MβCD, the change was revealed by real-time AFM immediately. Treatment with MβCD at 3 min and 14 min leads to apparent change in the Golgi membranes. As shown in Fig. 4B and 4E, some proteins in the membranes were eliminated, followed by the appearance of small pits. The average height of Golgi membranes also slightly decreased because some proteins in the membranes were removed by cholesterol depletion (see cross-section analysis in Fig. 4D). With the increase of incubation time, the major change takes place in the membrane at 22 min (Fig. 4F), in which there are only branched membrane patches on the surface, indicating that more lipid rafts were destroyed by MβCD. The eroded domains in the Golgi membrane, or called indentations pointed by the blue arrows in Fig. 4F, are irregular in shape. As shown in Fig. 4I, the size of the indentations varies from 20 nm to 200 nm, which is consistent with that of lipid rafts measured by other methods [38].

Although the MβCD treatment had most protein particles embedded in the membrane disappeared, there were still some small transmembrane proteins existing in the membrane. This result indicates that the proteins in the membrane are mostly located in the raft domains, and small portion of proteins exist in the non-raft domains.

### Observing Detergent Resistant Membranes in Golgi Membranes

The original definition of lipid rafts was from the region of detergent resistant membranes (DRMs). The non-ionic detergents, Triton X-100 as biochemical reagent is generally employed to prepare DRMs [39]. To explore whether the DRMs exist in Golgi leaflet membranes, we utilized time-lapse AFM to record the Golgi leaflet membranes images in PBS buffer before and after the treatment of Triton X-100.

**Figure 5:**
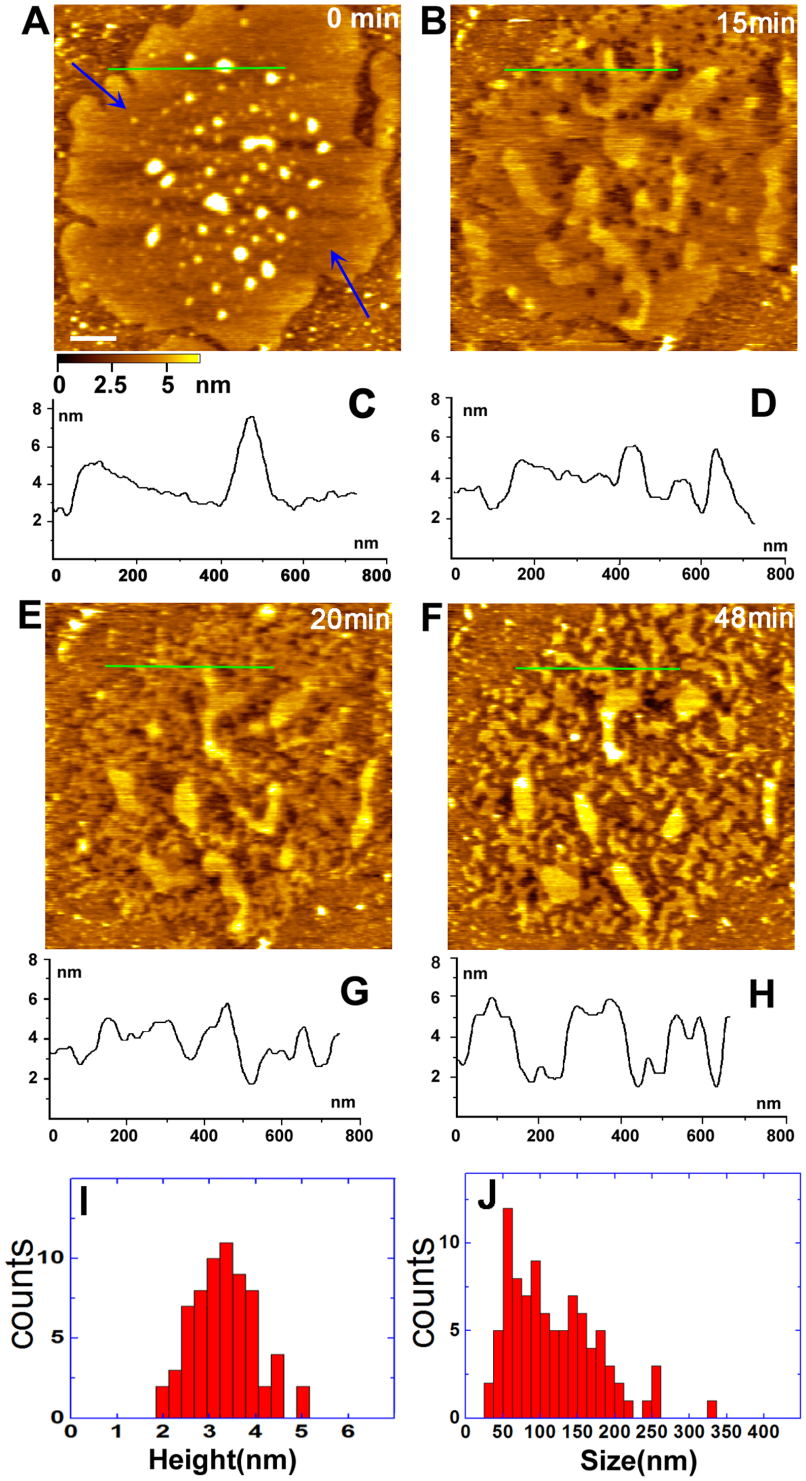
Real-time images of the Golgi inner leaflet membrane treated by Triton X-100. The images show the different stage after the addition of Triton X-100 for 0 min (A), 15 min (B), 20 min (E), and 48 min (F). Blue arrows in (A) point to the free lipid bilayer. Cross-section analysis along the green lines in images (A, B, E, F) are shown in (C, D, G, H), respectively. (I, J) Height and size distribution of the DRMs in the inner membrane obtained by Triton X-100 treatment. Scale bar in (A) is 200 nm.

Fig. 5A shows a typical AFM topographic image of the Golgi inner leaflet membrane with proteins in its center and free lipid bilayers on the edge before detergent extraction. The average height of free lipid bialyers indicated by blue arrows is 3.24±0.46 nm. To observe the DRMs, the Golgi leaflet membrane was treated by directly injecting 0.1% (v/v) Triton X-100 into the AFM sample chamber. The eroding process that the treated Golgi leaflet membrane changed with incubation time was recorded by real-time AFM.

After the addition of Triton X-100 for 15 min (Fig. 5B), some pits were visible at the membrane fraction and the proteins at the center of the membrane were removed, indicating that the non-resistant area of the membrane was initially solubilized. Subsequently, the whole membrane changed greatly at 20 min (Fig. 5E), accompanying with the irregular perforations observed. At 48 min (Fig. 5F), the membrane is further eroded by Triton X-100; as a result, some irregular membrane patches were produced. With prolonging incubation time, the shape of membrane patches remains unaltered (data not shown), indicating that these membrane patches are resistant to the Triton X-100 and can be considered as DRMs. The heights of the DRMs are mainly in the range of 2 nm to 5 nm with the average at about 3.30±0.66 nm (Fig. 5I). The sizes of DRMs are in the range of 20 nm to 350 nm with the major distribution from 50 nm to 200 nm (Fig. 5J). Taken together, these results indicate that the DRMs exist in the Golgi membrane. To our knowledge, for the first time, the DRMs of Golgi membrane are directly visualized under quasi-native conditions.

## Discussion

The Golgi apparatus serves as the central organelle and sorting station in living cells. The structure, molecular composition and dynamics of Golgi apparatus are essential for its functions. Owing to the complexity of the Golgi apparatus and limited research approaches, key issues regarding its structure remain controversial [13]. AFM is a unique approach that can image biological specimens in quasi-native environment at nanometer-scale resolution, allowing to provide direct evidences and profound insights into the structure of Golgi apparatus.

In our experiment, we directly absorbed the Golgi membrane fractions on the APTES-mica for imaging. The mica has atomic flat surface, which has less impact on the morphology of biology samples [40]. APTES-mica enriched with amino groups is suitable for the Golgi apparatus to be tightly attached on the mica surface (Fig. 1A). We utilized the AAC AFM to directly image the Golgi membrane fractions. In the scanning process, the AFM tip gently touches the sample at the Z-direction, and there is almost no lateral force generated, which greatly reduces the damage on biological samples.

The protein orientation of Golgi apparatus is the basis of its multiple functions, such as membrane sorting and signaling. AFM results provide direct and near-native evidences to show how proteins and lipids are arranged in the Golgi membranes. Significantly, we found the asymmetry of Golgi membranes, i.e. the proteins are mostly located in the inner leaflet of membranes and the outer leaflet shows a smooth surface, which is supported by the EM results from the previous investigations [41], [42]. This asymmetrical structure of Golgi apparatus could be fundamental to reveal how the polarized Golgi vesicles exchange the cargos and the membrane fusion happens during sorting. Further studies about the structure of nucleated cell membranes and endo-membrane system are necessary to reveal the mechanism of vesicles transporting in living cells.

The structure of Golgi apparatus membranes obtained by AFM imaging is in accordance with that of erythrocyte membranes from our previous results, in which the outer leaflet of erythrocyte membrane is smooth, whereas the inner leaflet is rough with dense embedded proteins. Previous investigations by biochemistry method and EM have successfully explored that the similarity existed between the Golgi membrane and the plasma membrane [43]. Our results provide direct evidences for the fact that the Golgi membranes have the similar features with plasma membranes morphologically.

The characteristics of the dynamic microdomains in Golgi membranes at the molecular level are vital for exploring the structure-function relationship of the Golgi apparatus. AFM has been a powerful tool to directly visualize lipid rafts in the model membranes [44] and cell membranes [21]. Here, the dynamic microdomains (lipid rafts) in Golgi membranes are directly observed by in situ and real-time AFM at the high resolution. In our experiment, the treatment of TritonX-100 and MβCD as two opposite approaches directly confirmed the existence of lipid rafts in Golgi apparatus membranes, which can not be achieved by EM due to the limitation of imaging conditions, such as fixation, staining and freezing. From current opinions, there are lipid rafts in eukaryotic cell membranes. Previous work has suggested that there are lipid rafts flux between the cell membranes and the Golgi complex [45]. It is assumed that the lipid rafts flux is closely related to membrane traffic and signal transduction.

Our previous AFM results have demonstrated that lipid rafts existed in the erythrocyte membranes. However, the erythrocyte membranes and the Golgi membranes experience different processes after the treatment of MβCD and TritonX-100. The outer leaflet membrane of the erythrocyte is more sensitive to TritonX-100 and MβCD, while the inner leaflet membrane is relatively stable. In contrast, the inner leaflet membrane of the Golgi is fairly sensitive to Triton X-100 and MβCD treatment. We assume that the main reason accounting for the phenomenon may be that the proteins distribution in the erythrocyte membranes is denser than that in the Golgi membranes. Given the importance and uncertainty of lipid rafts, more work are necessary to further reveal their nature and related functions in the Golgi apparatus.

### Conclusions

We used in-situ AFM to investigate the Golgi architecture at molecular level under quasi-native conditions. The basic structure of Golgi apparatus - the Golgi stack, single Golgi cisterna, associated tubules and vesicles have been imaged. AFM images suggest that the Golgi outer leaflet membrane is smooth, while the inner leaflet membrane is rough with dense proteins. The real-time AFM was utilized to observe the effect of MβCD on the inner leaflet membrane, and image the DRMs of the Golgi inner leaflet membrane treated by Triton X-100 under near physiological conditions at the nanometer resolution, which adequately confirmed that lipid rafts exist in the Golgi membranes. The shapes of lipid rafts are irregular, and the sizes of lipid rafts display a broad distribution from 20 nm to 200 nm. Our results provide a profound insight into the structure-function relationship of the Golgi complex at the molecule level, and would open up an exciting way for the ultrastructural analysis of mammalian endo-membrane systems.

## Materials and Methods

### Cell Culture and Preparation for Cell Homogenate

HeLa cells (parent from Shanghai institute of life science) were grown at 37°C in DMEM supplemented with 10% fetal bovine serum and in a humidified incubator with 5% CO_2_. The cells contained in five or six 90-mm diameter petri dishes with 80–90% confluence were used. The exponentially growing cells were collected by trypsinization and transferred to a 50 ml Falcon tube, and then washed twice with cold PBS by centrifugating at 1200 rpm for 5 min at 4°C. The tube containing cells was kept on ice. The cells were resuspended with 2 ml distilled water and removed into 5 ml flat tube, then crushed ice was added to the cell suspension to keep the cells at 0–4°C. The cells were lysed by vortexer for 5 min at 0–4°C.

### Isolation of Golgi-enriched Membranes from Cultured Hela Cells

The Golgi membrane fractions were isolated by sucrose density gradient centrifugation method from HeLa cells as described [46]. Gradient buffers were prepared as follows: buffers A–E were made up by the 0.5 M phosphate buffer, 2 M sucrose, 2 M MgC1_2_ and cold ultrapure water, and then the final PH was adjusted to 6.7. The sucrose concentration are 0 M for buffer A, 0.25 M for buffer B, 0.5 M for buffer C, 0.86 M for buffer D,1. 3 M for buffer E, respectively. A refractometer was necessary to check the refractiveindex of each buffer. The refractiveindex of homogenate was adjusted to that of buffer C (0.5 M sucrose) using buffer E. For the first gradient, 2 ml buffer D was placed into each ultraclear tube (MLS 50), then carefully overlaid by 1.5 ml homogenate. Finally, 0.6 ml buffer B was placed on the homogenate layer, and then the tubes were balanced within 0.01 g. The gradient was centrifuged at 144,000 g (MLS 50 rotor) for 60 min at 4°C. The cloudy band at the 0.5/0.86 M sucrose interface containing Golgi fractions was carefully removed from the gradient with an injector (1 ml). The refractiveindex of the obtained Golgi sample was adjusted to that of 0.25 M sucrose using buffer A. For the second gradient, 0.4 ml buffer E was added and followed by 0.8 ml buffer C into each tube, then 3.2 ml of diluted Golgi fraction was added. The gradient was centrifuged at 11,000 g (MLS 50) for 30 min at 4°C. The thin band at the 0.5/1.3 M sucrose interface was gently removed from the gradient with an injector. The aspirated Golgi membranes were mixed with buffer A to make the final volume to 4–5 ml. The mixture was centrifuged at 100,000 g (MLS 50) for 30 min at 4°C. The supernatant was discarded, and then 1 ml buffer A was added into the tube to mix the Golgi membranes. The diluted samples were aliquoted and stored at −20°C.

### APTES Functionalization Mica

APTES-mica substrate was prepared as described [47]. Briefly, after a desiccator was purged with argon for 2 min, 30 µl aminopropyltriethoxysilane (APTES, 99%, Sigma-Aldrich, St. Louis, MO) and 10 µl N,N-diisopropylethylamine (99%, Sigma-Aldrich, St. Louis, MO) were respectively placed into two small containers at the bottom of the desiccator, and the desiccator was purged with argon for 2 min again. Mica sheets were stripped on one side until smooth and immediately placed in the desiccator, then the desiccator was sealed off after being purged for further 3 min, leaving the mica exposed to the APTES vapor for about 1 h. The containers were removed, and the desiccator was purged again. The treated mica (APTES-mica) was stored in the sealed desiccator until used.

### Preparation of Glutaraldehyde Functionalization AP-mica and the Adsorption of Golgi Samples

200 µl of 1 mM glutaradehyde (grade I, Sigma-Aldrich) solution in water was pipetted onto APTES-mica, and incubated for 10 min [48]. The surface was rinsed with ultrapure water for 2∼3 times, then 30 µl Golgi samples was deposited onto the treated mica surface for 30 min. The surface was washed extensively with PBS for 2∼3 times to remove non-adsorbed Golgi membranes. The prepared sample was mounted into the SPM liquid cell containing PBS buffer and imaged immediately.

### Atomic Force Microscopy Imaging

AFM imaging was performed by 5500 AFM (Agilent Technologies, Chandler, AZ). The topographic images of Golgi apparatus were acquired by AAC mode AFM. Oxide-sharpened Si_3_N_4_ probes (Veeco, DNP-S) with a spring constant of (0.06 N/m) were used for imaging the soft Golgi apparatus sample. All the images were recorded with 512×512 pixels and at room temperature (21–25°C) in PBS buffer. The membrane height and particles size, as well the height and size of DRMs were measured using Picoscan 5.3.3 software (Agilent Technologies, Chandler AZ).

### Fluorescence Microscopy Imaging

Golgi-Tracker Red solution was diluted in the proportion of 1∶100 and incubated the solution at 37°C before use. After 30 µl of Golgi samples was deposited onto the treated APTES-slide surface for 30 min, the surface was washed extensively with PBS for 2∼3 times to remove non-adsorbed Golgi membranes. 50 µl of Golgi-Tracker Red solution was dropped onto the APTES-slide to attach the Golgi samples for 30 min in the dark condition. After the slide surface was extensively washed with PBS for 2∼3 times, the Golgi samples were imaged in the PBS buffer with a Nikon EclipseTi Series Microscope.

## Supporting Information

Figure S1 **Western blot analysis of the Golgi membrane fractions.** The existence of the Golgi membrane fractions was confirmed by Western blot analysis using anti-β-1,4-Galactosyltransferase.The isolated Golgi membrane fractions and cells were dissolved in lysis buffer (150 mM NaCl, 20 mM Tris, 5 mM EDTA pH 7.5, 1% Triton X-100, and supplemented with 1 mM PMSF), respectively. Fifty micrograms of proteins were resolved in 10% SDS-PAGE, and transferred to NC membranes. After blocking with 5% (w/v) nonfat milk and washing in Phosphate-buffered saline-Tween solution, membranes were incubated with primary goat anti-β-1,4-Gal-T1 polyclonal antibody (Santa Cruz, 1∶500) for 2 h, washed and then incubated with secondary rabbit anti-goat IgG antibody conjugated to Alkaline Phosphatase (Sigma, 1∶10000) and detected using an BCIP/NBT Liquid Substrate System (Sigma). The existence analysis of Golgi membrane fractions isolated from HeLa cells by Western blot analysis. Cell lysates: total HeLa cells lysates from lysis buffer; Pure Golgi: Golgi membrane fractions from HeLa cells isolated by ultracentrifugation method. The Golgi maker for β-1,4-Galactosyltransferase was little expressed in the cell lysates, but abundantly expressed in the Golgi membrane fractions. The Western blotting result indicates that the yields of the Golgi membranes were high.
(TIF)

Figure S2 **Fluorescence imaging of Golgi apparatus.** To further verify the existence of the Golgi fractions in isolated samples, we observed the samples dyed with the Golgi-tracker Red (specific fluorescent dye of the Golgi complex) by fluorescence microscopy. (A) Fluorescent image of Golgi membrane fractions (red) labeled with Golgi-tracker Red. The Golgi stack (in blue box) and the Golgi vesicles or single Golgi cisternae (in green box) are imaged. Scale bar is 5 µm. (B) Control experiment. The Golgi-tracker Red was dropped onto the APTES-slide following the washing step by PBS buffer. There is no obvious signal observed. The scale bar is 5 µm.
(TIF)

Figure S3 **AFM image of the cell membrane, ER and mitochondria.** To further confirm that the Golgi membrane fractions are different from the other cellular organelles, we isolated the cell membrane, ER and mitochondria, respectively. The prepared cell membrane, mitochondria and ER were imaged by AFM in PBS solution. Apparently, the Golgi apparatus is distinguished from the cell membrane, mitochondria and ER in morphology. (A) AFM image of Hela cell membrane. The scale bar is 1 µm. (B) AFM image of the ER membrane isolated from Hela cells. The scale bar is 200 nm. (C) AFM image of single mitochondrion. The scale bar is 500 nm.
(TIF)
