# Supplementary figures and images for: The Asymmetrical Structure of Golgi Apparatus Membranes Revealed by In situ Atomic Force Microscope
Source: PLoS One. 2013 Apr 16;8(4):e61596. doi: 10.1371/journal.pone.0061596 (PMC3628984; doi:10.1371/journal.pone.0061596)

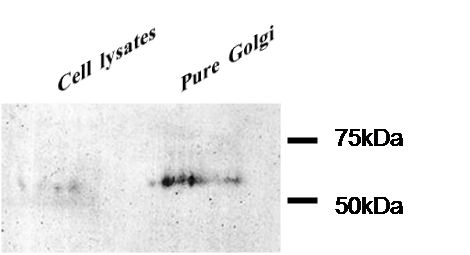

Supplement: Figure S1 — Western blot analysis of the Golgi membrane fractions. The existence of the Golgi membrane fractions was confirmed by Western blot analysis using anti-β-1,4-Galactosyltransferase.The isolated Golgi membrane fractions and cells were dissolved in lysis buffer (150 mM NaCl, 20 mM Tris, 5 mM EDTA pH 7.5, 1% Triton X-100, and supplemented with 1 mM PMSF), respectively. Fifty micrograms of proteins were resolved in 10% SDS-PAGE, and transferred to NC membranes. After blocking with 5% (w/v) nonfat milk and washing in Phosphate-buffered saline-Tween solution, membranes were incubated with primary goat anti-β-1,4-Gal-T1 polyclonal antibody (Santa Cruz, 1∶500) for 2 h, washed and then incubated with secondary rabbit anti-goat IgG antibody conjugated to Alkaline Phosphatase (Sigma, 1∶10000) and detected using an BCIP/NBT Liquid Substrate System (Sigma). The existence analysis of Golgi membrane fractions isolated from HeLa cells by Western blot analysis. Cell lysates: total HeLa cells lysates from lysis buffer; Pure Golgi: Golgi membrane fractions from HeLa cells isolated by ultracentrifugation method. The Golgi maker for β-1,4-Galactosyltransferase was little expressed in the cell lysates, but abundantly expressed in the Golgi membrane fractions. The Western blotting result indicates that the yields of the Golgi membranes were high. (TIF) [file pone.0061596.s001.tif]

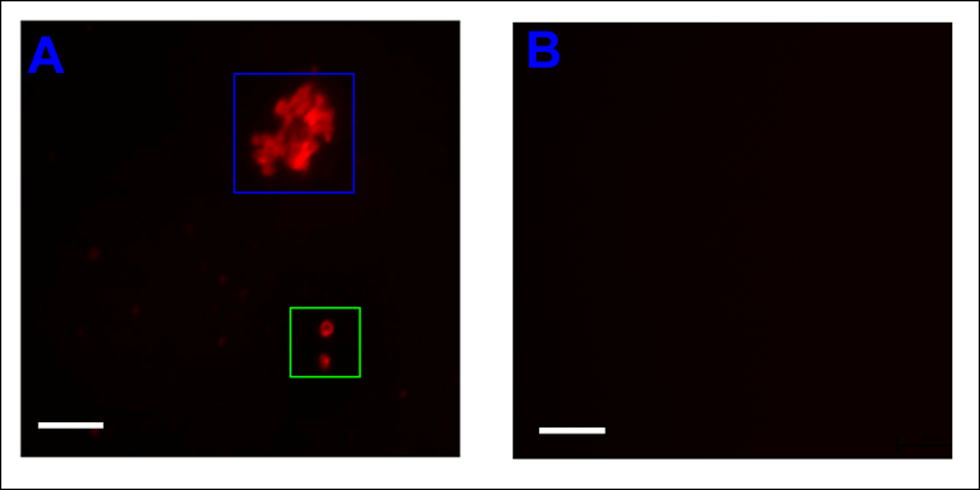

Supplement: Figure S2 — Fluorescence imaging of Golgi apparatus. To further verify the existence of the Golgi fractions in isolated samples, we observed the samples dyed with the Golgi-tracker Red (specific fluorescent dye of the Golgi complex) by fluorescence microscopy. (A) Fluorescent image of Golgi membrane fractions (red) labeled with Golgi-tracker Red. The Golgi stack (in blue box) and the Golgi vesicles or single Golgi cisternae (in green box) are imaged. Scale bar is 5 µm. (B) Control experiment. The Golgi-tracker Red was dropped onto the APTES-slide following the washing step by PBS buffer. There is no obvious signal observed. The scale bar is 5 µm. (TIF) [file pone.0061596.s002.tif]

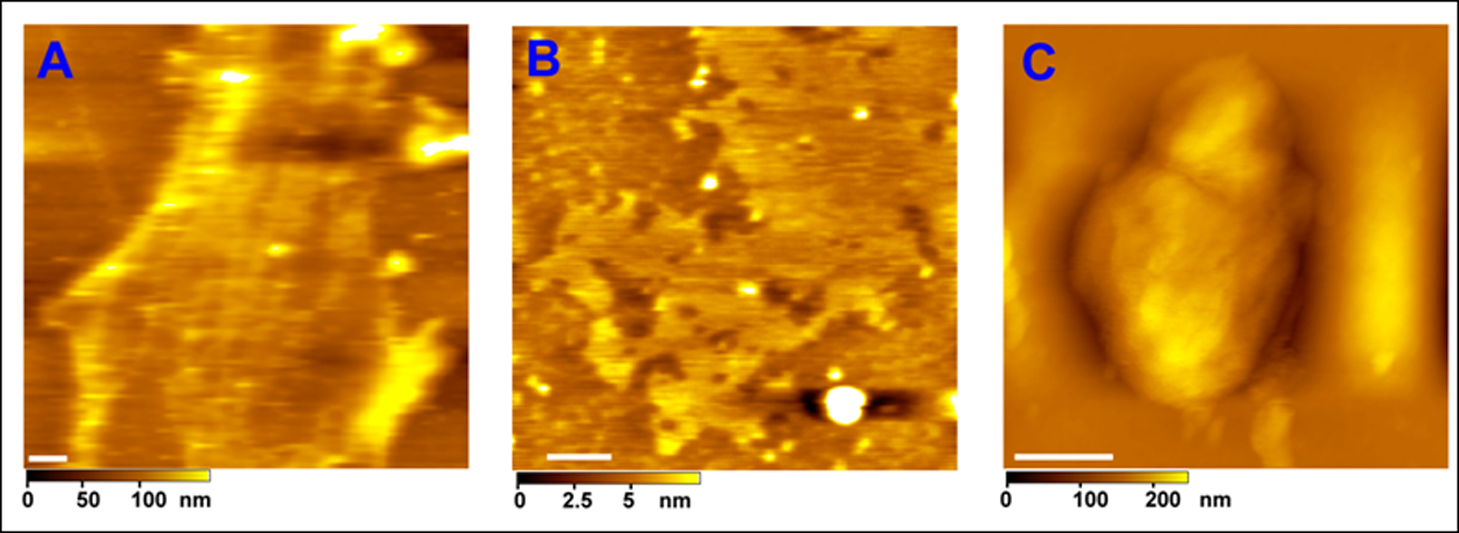

Supplement: Figure S3 — AFM image of the cell membrane, ER and mitochondria. To further confirm that the Golgi membrane fractions are different from the other cellular organelles, we isolated the cell membrane, ER and mitochondria, respectively. The prepared cell membrane, mitochondria and ER were imaged by AFM in PBS solution. Apparently, the Golgi apparatus is distinguished from the cell membrane, mitochondria and ER in morphology. (A) AFM image of Hela cell membrane. The scale bar is 1 µm. (B) AFM image of the ER membrane isolated from Hela cells. The scale bar is 200 nm. (C) AFM image of single mitochondrion. The scale bar is 500 nm. (TIF) [file pone.0061596.s003.tif]
